# Enhancing the Bioconversion of Azelaic Acid to Its Derivatives by Response Surface Methodology

**DOI:** 10.3390/molecules23020397

**Published:** 2018-02-13

**Authors:** Nurshafira Khairudin, Mahiran Basri, Hamid Reza Fard Masoumi, Shazwani Samson, Siti Efliza Ashari

**Affiliations:** 1 Department of Chemistry, Faculty of Science, Universiti Putra Malaysia, Serdang 43400, Selangor, Malaysia; nurshafirakhairudin@yahoo.com (N.K.); dorawanie_89@yahoo.com (S.S.); 2 Department of Biomaterials, Iran Polymer and Petrochemical Institute, Tehran 14977-13115, Iran

**Keywords:** azelaic acid, anti-acne, enzymatic reaction, Novozym 435, response surface methodology (RSM)

## Abstract

Azelaic acid (AzA) and its derivatives have been known to be effective in the treatment of acne and various cutaneous hyperpigmentary disorders. The esterification of azelaic acid with lauryl alcohol (LA) to produce dilaurylazelate using immobilized lipase B from *Candida antarctica* (Novozym 435) is reported. Response surface methodology was selected to optimize the reaction conditions. A well-fitting quadratic polynomial regression model for the acid conversion was established with regards to several parameters, including reaction time and temperature, enzyme amount, and substrate molar ratios. The regression equation obtained by the central composite design of RSM predicted that the optimal reaction conditions included a reaction time of 360 min, 0.14 g of enzyme, a reaction temperature of 46 °C, and a molar ratio of substrates of 1:4.1. The results from the model were in good agreement with the experimental data and were within the experimental range (R^2^ of 0.9732).The inhibition zone can be seen at dilaurylazelate ester with diameter 9.0±0.1 mm activities against *Staphylococcus epidermidis* S273. The normal fibroblasts cell line (3T3) was used to assess the cytotoxicity activity of AzA and AzA derivative, which is dilaurylazelate ester. The comparison of the IC_50_ (50% inhibition of cell viability) value for AzA and AzA derivative was demonstrated. The IC_50_ value for AzA was 85.28 μg/mL, whereas the IC_50_ value for AzA derivative was more than 100 μg/mL. The 3T3 cell was still able to survive without any sign of toxicity from the AzA derivative; thus, it was proven to be non-toxic in this MTT assay when compared with AzA.

## 1. Introduction

Azelaic acid (AzA; HOOC-(CH_2_)_7_-COOH), also known as 1,7-heptanedicarboxylic acid, is a naturally occurring saturated C9-dicarboxylic acid. Azelaic acid possesses antimicrobial, anti-inflammatory, and comedolytic actions with a well-established role in the treatment of acne vulgaris [1]. Currently, Azelaic acid is available in the market as a 20% azelaic acid cream for the treatment of acne vulgaris (AV) when applied twice daily, and it has been approved for use by the Food and Drug Administration (FDA) [2]. These formulations have been shown to be effective in the treatment of inflammatory (nodulocystic, papulopustular, and nodular) acne and comedonal acne, as well as in various cutaneous hyperpigmentary disorders in pharmacological applications. However, azelaic acid is limited in terms of cosmaceutical and pharmaceutical application because it has a high melting point, poor solubility properties, and requires a high dosage. The latter increases the incidence of side effects such as local irritation, stinging, burning, and redness of the skin associated with exposure to high levels of undissolved, dispersed AzA that has an inherent low pH [3].

A derivative of azelaic acid (dilaurylazelate ester) with even better characteristics than the original starting material may be produced through the modification to azelaic acid, especially the efficacy of azelaic acid derivatives in the treatment of various dermatological and cosmetic disorders, which involve inflammation and bacterial and fungal infection [4]. Modification of azelaic acid by attachment through covalent ester linkages and converting at least one of the carboxylic acid groups into the ester group has enhanced not only the technical property of azelaic acid but also the functional performance of the resulting modified azelaic acid [4].

The modified AzA as a prodrug showed the efficacy of a relatively low dosage of AzA derivatives in the treatment of various cutaneous hyperpigmentary disorders and acne vulgaris [4]. The prodrug has been extensively improved by modifying the drug’s hydrophilic–lipophilic balance to improve the bioavailability of many drugs. Many prodrugs were synthesized, resulting in successfully improved bioavailability [5]. To improve the efficiency of drugs by the dermal route would be to manipulate the physicochemical properties to increase the rate of diffusion into the skin barrier. A promising approach in this respect is the development of prodrugs [6,7]. Prodrugs can facilitate the transfer of a drug into or across the skin through the addition of a cleavable chemical group that typically increases a drug’s lipophilicity. Because the prodrug approach is based on altering a drug’s structure, prodrugs usually produce no skin irritation [8].

Hsieh et al. (2012) also introduced a co-drug of azelaic acid and conjugated hydroquinone to enhance topical skin targeting and decrease penetration through the skin. Their studies demonstrated that esterification of azelaic acid incorporated with hydroquinone contains two hydroxyl functionalities, which allow the formation of an ester co-drug using Dimethyl cyclohexylcarbodiimide (DCC) and 4-dimethyl aminopyridine (DMAP),which is chemical catalyst that obtains only 52% yield [9]. Ethanolic ester of azelaic acid was synthesized by Manosroi et al. (2016) to study its impact on cytotoxicity activity and its effect on azelaic acid diffusion through the silicone membrane [5].

Production of such esters from AzA by esterification reactions requires either a chemical catalyst or an enzyme. A chemical catalyst normally requires high pressure and high temperatures, as well as corrosion-resistant equipment, which often results in undesired by-products. The preparation of the fatty acid substrates, for example, produces many impurities, producing a dark-coloured product. Subsequent downstream processes such as distillation and bleaching are needed to obtain lighter-coloured and purer products [10]. In addition, due to their high capability of solvating the raw materials, most of chemical syntheses employ toxic organic solvents as reaction media. The product esters containing toxic solvent residue are not permitted in the pharmaceutical industries [11].

Lipases are the most versatile biocatalysts and have been extensively employed for the direct synthesis of many esters [12]. Lipases contain a helical oligopeptide unit called “lid” or “flap” that shields the active site. The active site is composed of serine, histidine, aspartate, and a carboxylic acid residue (aspartic or glutamic acid). The lid, upon interaction with a hydrophobic interface such as a lipid droplet, undergoes a conformational change in such a way that exposes the catalytic site to free access of the reaction substrate. However, *Candida antarctica* lipase B (CalB) possesses an open active site and does not have a real lid. Therefore, CalB does not display any interfacial activations or conformational changes in which a lid region removes to uncover the active site. Among the lipases, *Candida antarctica* lipase B has been reported to show a high catalytic activity for esterification of dicarboxylic acids [13].

The synthesis of AzA derivatives was carried out using immobilized *Candida antarctica* lipase B as a biocatalyst. Zisis et al. (2015) reported that CalB was recently reclassified as Pseudozymaantartica lipase B (PalB) [14]. This lipase has been shown in numerous publications to be a particularly efficient enzyme, catalyzing a great number of different reactions including both regio- and enantio-selective synthesis. This lipase demonstrates that enzymes can be not only very effective but also highly robust tools within organic synthesis [15]. In this study, the effect of different variables on the degree of conversion of AzA was investigated using RSM and the optimization of the reaction variables including the reaction times (90, 180, 270, 360, and 450 min), enzyme amounts (0.05, 0.15, 0.25, 0.35, and 0.45 g),reaction temperatures (40, 46, 52, 58, and 64 °C), and substrates molar ratios (1:3,1:4.5, 1:6, 1:7.5, and 1:9 mole, AzA:LA) is reported. The impact of cytotoxicity activity AzA derivatives on the fibroblasts cell line (3T3), and its antibacterial activity against on the bacteria *Staphylococcus epidermidis S273,* was studied.

## 2. Results and Discussion

### 2.1. Identification of Dilauryl Azelate Ester

**Figure 1:**
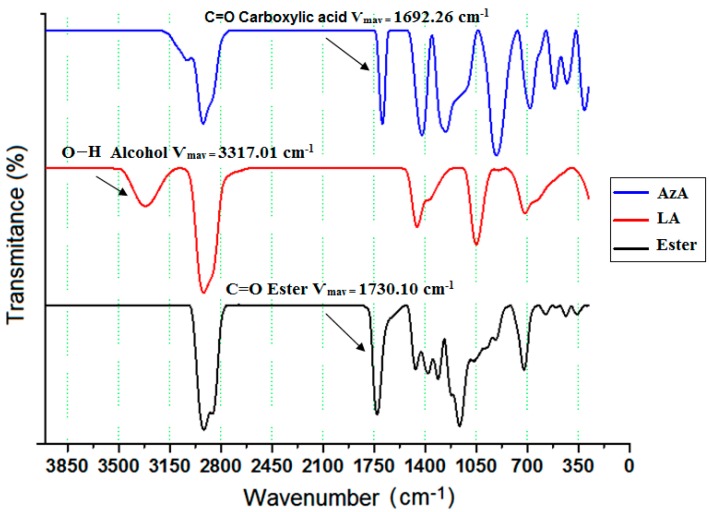
Infrared spectrum of dilaurylazelate ester.

The identification of the dilaurylazelate ester was determined by TLC, FT-IR, GCMS, and NMR. Thin layer chromatography (TLC) was used to confirm the production of the dilaurylazelate ester in the initial biocatalyst screening. The least polar component, dilaurylazelate ester (R_f_ = 0.57), was eluted first, followed by lauryl alcohol (R_f_ = 0.25). Azelaic acid with a higher polarity was the last component eluted (R_f_ = 0), as it was absorbed onto the stationary phase more than the dilaurylazelate ester and lauryl alcohol; thus, it moved through the stationary phase more slowly. Figure 1 illustrates the FT-IR spectra of azelate acid, lauryl alcohol, and dilaurylazelate ester. The FTIR spectra of azelaic acid showed an absorption at Ѵ_max_ 1692.26 cm^−1^ (C=O carboxylic acid), whereas lauryl alcohol had an absorption at Ѵ_max_ 3317.01 cm^−1^ (O-H alcohol stretch), and the product had an absorption at Ѵ_max_ 1730.10 cm^−1^ (C=O ester), indicating the successful formation of the ester.

The gas chromatography mass spectroscopy (GCMS) spectrum of the dilaurylazelate ester showed a molecular ion peak at *m*/*z* 525 [M + H]^+^ with base peak = 171, which matched the molecular weight of the dilaurylazelate ester (C_33_H_64_O_4_). ^1^H NMR and ^13^C NMR indicated the presence of dilauryl alcohol linked to azelaic acid and the respective carbon and hydrogen positions. ^1^H NMR ((CD_3_)_2_CO, TMS): 4.05 (t, 4H, *J* = 10Hz), 2.30 (t, 4H, *J* = 10 Hz), 1.62 (m, 8H), 1.31 (m, 42H), 0.90 (t, 6H, *J* = 10 Hz). ^13^C NMR: 13.49, 22.46, 24.78, 25.80, 28.58, 28.84, 28.96, 29.07, 29.21, 29.41, 29.44, 29.49, 29.51, 31.77, 33.75, 63.69, 172.76.

### 2.2. Mathematical Model and Analysis of Variance (ANOVA)

The efficacy of Novozym 435 was tested in the esterification of azelaic acid with lauryl alcohol by selecting four variables, as shown in Table 1. The experiments were programmed as per the CCRD (central composite rotatable design) layout, and the degree of conversion of AzA was selected as the response variable ((design expert software version 7.0) Stat-Ease, Minneapolis, MN, USA) involving 30 different combinations of the coded variables (Table 2). In order to demonstrate reproducibility, each experimental run was conducted in triplicate. The optimal process conditions corresponding to maximum dilaurylazelate ester yields and minimum enzyme amounts were then computed using mathematical models in the Design Expert software. Optimization of the process parameters by RSM techniques has been reported by several research reports [16,17,18].

The experimental conversion values at each point according to the experimental design are shown in Table 2. Based on the central composite rotatable design and results of the experiments, the coefficients of the regression equation (Equation (1)) were calculated using the Design Expert 7.0 software (Stat-Ease, Minneapolis, MN, USA). The following second-order polynomial equation was established to obtain the degree of conversion of AzA. Where A is enzyme amount, B is the reaction time, C is the reaction temperature, and D is the molar ratio of substrates. The positive sign in front of the terms indicates a synergistic effect, whereas the negative sign indicates an antagonistic effect. Negative values of a coefficient estimate denote a negative influence of parameters on the reaction yield.

(1) $$\begin{array}{l} Y(\mathrm{Degree}\;\mathrm{of}\;\mathrm{Conversion}\%)\\ \;=+86.24+0.11\;A+3.83\;B+1.29\;C-1.63\;D+0.64\;\mathrm{AB}+\;1.16\;\mathrm{AC}\\ \;-\;1.79\;\mathrm{BC}-0.89\;\mathrm{BD}+1.75+0.65\;B^{2}+0.74\;C^{2}-0.92\;D^{2} \end{array}$$

The mathematical model found after fitting the function to the data can sometimes not satisfactorily describe the experimental domain studied. The more reliable way to evaluate the quality of the model fitted is by applying analysis of variance (ANOVA). ANOVA analysis was necessary in order to determine whether or not the second-order polynomial model was significant. Table 3 indicated that the quadratic polynomial model was statistically significant (*p*-value < 0.0001) and adequate to represent the actual relationship between the response (degree of conversion, %) and the significant variables. The coefficient of determination (R^2^) and the R^2^_adj_ of the model obtained were 0.9732 and 0.9542, respectively, which indicated that the accuracy of the polynomial model was adequate. The “Lack of Fit *F*-value” of 0.7794 implied the lack of fit was not significant relative to the pure error.

The *p*-value was also used to check the significance of each coefficient. The smaller the *p*-value, the more significant was the corresponding coefficient. It can be seen from the table that the coefficients of B, C, D, AB, AD, BD, CD, A^2^, B^2^, C^2^, and D^2^ were statistically significant, with *p*-values below 0.05. The most significant variable was the reaction time (*p*-value of 0.0001 ≪ 0.05), followed by the molar ratio of substrates and reaction temperature. The immobilized catalyst dosage, as one of main variables, did not show any significant effect, but when multiplied by itself (A^2^) it played a very important role in the model (*F*-value 75.60, *p*-value 0.0001).

**Figure 2:**
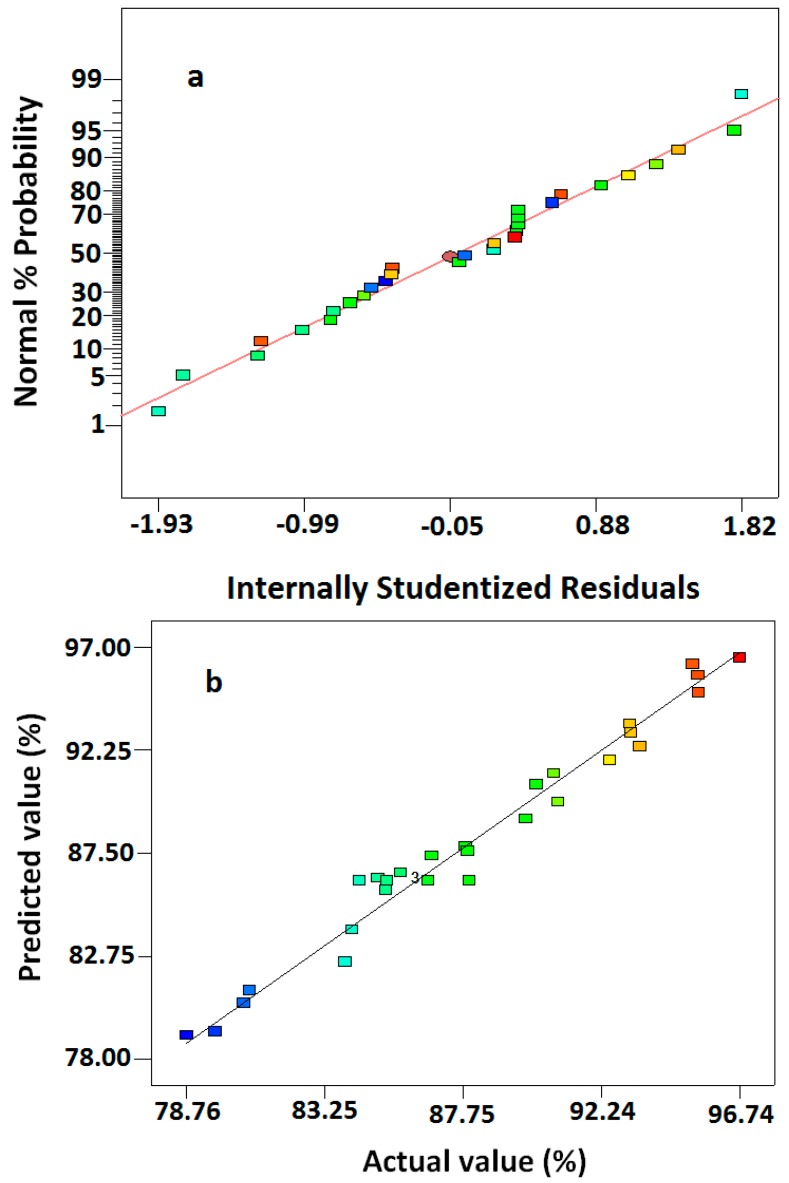
(**a**) The residual plot of runs from central composite rotatable design; (**b**) scatter plot of predicted conversion% value versus actual conversion% value.

The residual plot is an important diagnostic tool to detect the systematic departures from the assumptions used in developing the regression equations [19]. As shown in Figure 2a, the data points on this plot were close to a straight line, confirming the assumption of normal distribution and the independence of the residuals. Figure 2b shows the relationship between the actual and predicted values for the conversion of AzA. The actual values represented the measured response data for a particular run, and the predicted values were generated by the model. As observed in Figure 2b, the predicted values correlated with the actual values with an R^2^ of 0.9732. This indicated the accuracy of the model in determining the correlation between the conversion and the independent reaction variables.

### 2.3. Mutual Effects of Process Parameters

**Figure 3:**
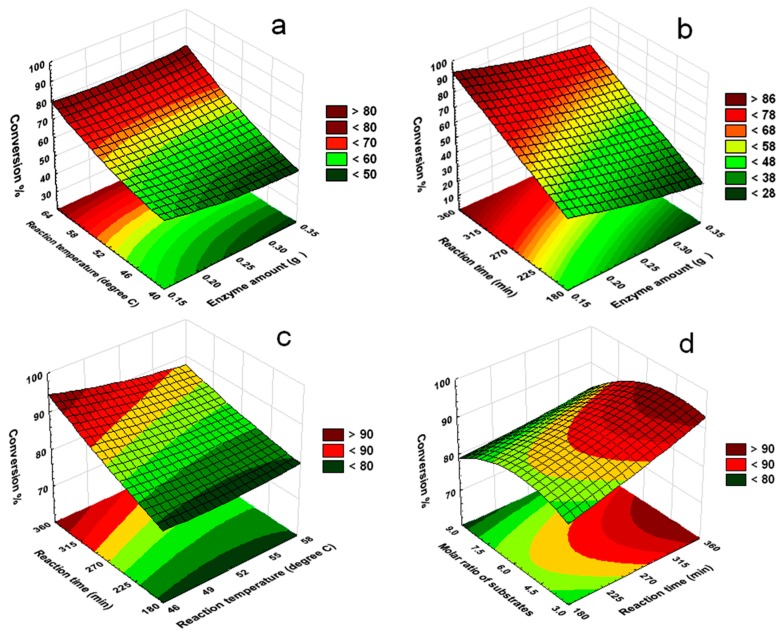
Response surface plots: (**a**) reaction temperature (°C) versus enzyme amount (g); (**b**) reaction time (min) versus enzyme amount (g); (**c**) reaction time (min) versus reaction temperature (°C); (**d**) reaction time (min) versus molar ratio of substrates (AzA:LA) (mole) on the percentage conversion as a response.

The terms in Equation (1) showed that the interactions between variables had significant effects on the degree of conversion% of the enzyme-catalyzed reaction. Therefore, the interactions between variables could be further investigated to clarify their significance and importance for a more comprehensive optimization, instead of studying single variables individually. Figure 3a shows the interaction between enzyme amounts and reaction temperatures on the degree of AzA conversion. As reaction temperature increased, the degree of AzA conversion correspondingly increased till a further extension in the temperature variable was not possible due to the boiling point of the solvent (*n*-Hexane, *bp* ≈ 68 °C). However, denaturation of the enzyme could also occur at higher reaction temperatures [20,21].

Figure 3b shows the effect of reaction times and enzyme amounts on the degree of AzA conversion. A response surface plot for the interaction between enzyme amounts and reaction times was generated with the other variables kept constant. The data obtained proved that the amount of enzyme did not significantly affect the degree of conversion%. However, at any given enzyme amount between 0.15–0.35 g, the degree of conversion% increased with increased reaction time.

The predicted response surface plots for the interaction of reaction time and reaction temperature are presented in Figure 3c. At any given reaction time from 90 to 450 min and any given reaction temperature from 40 to 60 °C, the percentage of conversion increased to maximum value. The figure showed that there was an increase in the percentage of conversion with an increase in both variables, but a reaction with longer reaction time and lower reaction temperature could increase the conversion of ester to a higher percentage. This indicated that the conversion of ester was greatly affected by both reaction time and reaction temperature.

Figure 3d depicts the effect of reaction time and molar ratio of substrates on the percentage of conversion when the other variables were kept constant. It can be observed that the maximum conversion of AzA was achieved when the reaction time increased while the molar ratio of substrates decreased. From the data obtained, it was expected that very low levels of esterification were observed at shorter reaction times. Reactions with longer reaction time could allow for an increased conversion of ester. It was also observed that the degree of conversion of AzA increased until the substrate molar ratio reached a 1:4.1 molar ratio. This observation was also similar to data obtained by Basyaruddin et al. (2008) who reported the optimized enzymatic synthesis of dilauryladipate ester using response surface methodology [22].

### 2.4. Reaction Optimization and Model Validation for Dilauryl Azelate Ester

Optimum reaction conditions are needed to achieve the desired degree of conversion of AzA. Optimum conditions allow for the highest degree of conversion and have the lowest enzyme amounts, faster reaction times, and lower reaction temperatures. It would be favorable to obtain the highest conversion percentage for better productivity and improved efficiency, while the lowest possible enzyme amounts were used in order to save costs due to the economic concerns involved. With reference to Table 2, experiment number 12 yielded a higher degree of conversion compared to the optimum conditions, but the amount of enzyme used was also higher (0.25 g). To achieve the maximum conversion of substrate, it was preferable to use the minimum enzyme amount [23,24]. It is impractical to increase the conversion percentage by utilizing a higher amount of enzyme and a heavier cost of production, as immobilized lipase (Novozym 435) is more expensive than the other substrates; thus, it was desirable that a higher reaction conversion was attained by a lower amount of enzyme. Moreover, lower reaction temperatures and shorter reaction times were considered in the optimization process to avoid possible enzyme denaturation.

Optimization was carried out by identifying the optimum conditions for a higher reaction conversion percentage as shown in Table 4. From multivariate analysis, the optimum reaction variables were: 360 min of reaction time, 0.14 g of enzyme, a reaction temperature of 46 °C, and a mole of substrates molar ratio (AzA:LA) of 1:4.1. The experimental reaction gave a reasonable percentage conversion of 95.38%. The experimental data confirmed the model validity, and the data achieved was quite close to the predicted value (96.32%), implying that RSM based on CCRD experimental design was an accurate and reliable method to optimize the reaction conditions.

In addition, five random reaction conditions were prepared to validate the model. The adequacy of the final model was checked by comparing the predicted values with the experimental values. The recommended optimum conditions were also conducted to verify the optimum conversion percentages that were predicted by the model. The percentage of residual standard error (RSE%) was calculated for the response.

### 2.5. Evaluation of the Importance of the Variables on the Reaction Conversion

**Figure 4:**
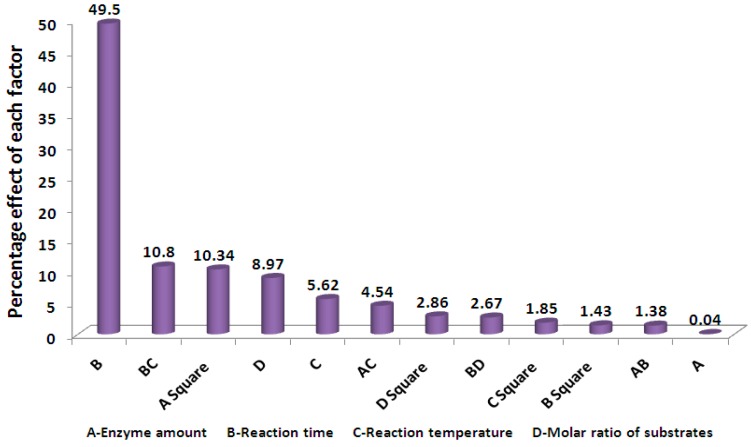
Pareto graphic analysis describes the effect of each of the variables (enzyme amount, reaction time, reaction temperature and molar ratio of substrates) in percentages (%).

The important influence of effective variables on the conversion percentages was illustrated by Pareto analysis in Figure 4. The Pareto analysis describes the effect of each variable, in percentages, on the response according to the coefficient of the coded variable in Equation (2) [18,25].

(2) $$P_{i}=\left(\frac{b_{i}^{2}}{\sum b_{i}^{2}}\right)\times 100\;\left(i\neq 0\right)$$

The most important factor that affected the conversion percentage was the reaction time (B) at 49.50%, followed by the interaction between reaction times and reaction temperatures (BC) at 10.80% and the square form of the enzyme amount factor (A^2^) at 10.34%.

### 2.6. Antibacterial Assay of Dilauryl Azelate Ester

The mechanism of the reported bacterial activity of AzA remains to be confirmed. However, effects on enzyme activities, macromolecular synthesis, and intracellular pH have been suggested. No bacterial resistances to azelaic acid have as yet been reported. The pH is crucial for the bacteriological activity of AzA, which is only active at specific values of pH 4–6 Thiboutot et al., 2008. Normal skin pH has been estimated to be 5.0–6.0. A pH of 5.6 was chosen as representative, following the lead of others who have conducted tests similar to those in the present study.

**Figure 5:**
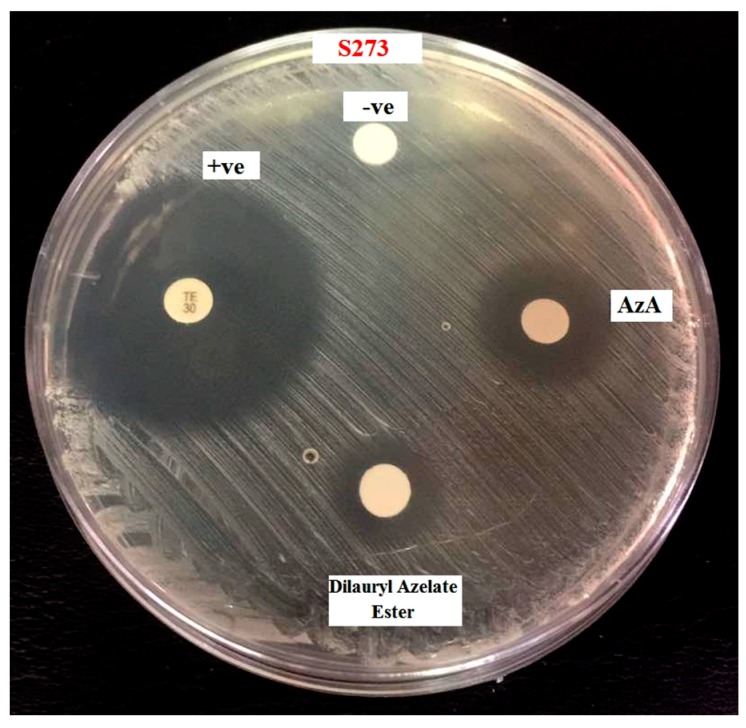
The inhibition zone of AzA and dilaurylazelate ester on bacteria *Staphylococcus epidermidis S273*, (−ve is Acetonitrile and +ve is Standard (Streptomycin)).

The antibacterial activity against the pathogen bacteria *Staphylococcus epidermidis S273* of AzA and dilaurylazelate ester was estimated by the disc and well diffusion agar methods at pH 5.6, and the results are shown in Table 5. The size of the inhibition zone indicated the antibacterial effect of AzA and dilaurylazelate ester [1]. Figure 5 represented the inhibition zone of dilaurylazelate ester on bacteria *Staphylococcus epidermidis S273.* Based on the result obtained, it showed the inhibition zone can be seen at AzA with diameter 11.5 ± 0.1 mm, while the dilaurylazelate ester also gave the diameter inhibition zone 9.0 ± 0.1 mm, as well as AzA. This effect is dependent on the concentration of dilaurylazelate ester and AzA, as well as any putative condition causing a local increase in skin pH.

### 2.7. Cytotoxicity Assay of Dilauryl Azelate Ester

**Figure 6:**
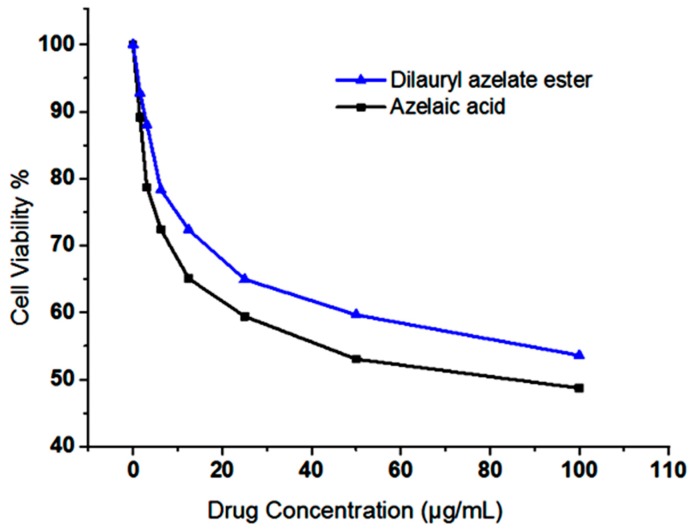
Comparison the toxicity effect of azelaic acid (AzA) and AzA derivatives (dilaurylazelate ester) on normal fibroblasts cell line (3T3). Data represented in percentage (%) of cell viability (IC_50_).

Normal fibroblasts cell line (3T3) was used to assess the toxicity of AzA and AzA derivative, which is dilaurylazelate ester. Concentration cytotoxicity of both compounds was evaluated by MTT colorimetric assay to test cellular response of the 3T3 cell (Figure 6). It was showed the comparison of IC_50_ (50% inhibition of cell viability) value for AzA and AzA derivative. IC_50_ value for AzA was 85.28 μg/mL, whereas AzA derivative was more than 100 μg/mL. Based on the result, IC_50_ value of AzA derivative did not reach up to the highest concentration tested. (Gad, 2009) reported that IC_50_ value lower than 10 μg/mL is very toxic, while 10–100 μg/mL is toxic. In this study, the cells were still able to survive without any sign of toxicity for dilaurylazelate ester; thus, it was proven to be non-toxic in this MTT assay compared with AzA and is safe to be used in pharmaceutical applications [26].

## 3. Materials and Methods

### 3.1. Materials

Lauryl alcohol and azelaic acid were obtained from Merck (Darmstad, Germany). Immobilized *Candida antarctica* lipase B (Novozym 435) was purchased from Novo Nordisk Industries (Bagsvaerd, Denmark). All solvents and reagents were of analytical high-performance liquid chromatography (HPLC) grade. *n*-Hexane, chloroform, dichloromethane, acetone, ethanol, acetonitrile, and diethyl ether obtained from J.T.Baker (Center Valley, PA, USA) was used as the organic solvent. All other reagents used were of analytical grade.

### 3.2. Method

#### 3.2.1. Enzymatic Esterification of Azelaic Acid and Analysis of Samples

In a typical experiment, azelaic acid (1.59 mmol), a proportional amount of lauryl alcohol, and different amounts of Novozym 435 in *n*-hexane (5 mL) were placed in 10 mL screw-capped glass vials. The 3Å molecular sieves (Merck) (0.03g) that were added in the reaction mixture helped to remove water from organic media. The mixture was stirred (150 rpm) in a horizontal water bath shaker. When the AzA substrate was entirely converted according to the adjusted reaction times, the mixture was filtered to remove the enzyme.

A titration method using 0.1 M NaOH solution was applied to determine the percentage of AzA conversion. The AzA conversions were calculated from the titration volumes of the NaOH solution for the reaction samples, with enzyme (test) and without enzyme (control). The product formed was expressed as equivalent to the conversion of acid [27,28]. The percentage of the degree of conversion of AzA was calculated by the consumption of NaOH solution (0.1 M) for both the experiment and the control. The degree of conversion was determined using Equation (3).

For purification of the azelate ester, after termination of the reaction, the enzyme was filtered and the solvent removed by evaporator under reduced pressure. Product in the remaining mixture were separated via silica gel (Kieselgel 60, Merck, particle size 0.063–0.200 mm) column chromatography (15 cm × 20 mm) using a chloroform/dichloromethane (95/5, *v*/*v*) mixture as eluent. A sample made up of 1:1 (*w*/*w*) ratio of silica gel and the free solvent reaction mixture was deposited at the top of the column previously equilibrated with chloroform/dichloromethane (95/5, *v*/*v*) mixture. Five millilitre fractions were collected and tested using thin layer chromatography to identify the product-rich portions. Such fractions were pooled and the solvent evaporated by a rotary evaporator. The purity of product was then checked with TLC before FTIR, GCMS, and NMR.

A solution of dichloromethane:chloroform (5:95, *v*/*v*) was selected as the mobile phase for the thin-layer chromatography (TLC) analysis. Further identification of the ester formed was determined by FTIR (Perkin Elmer, Bridgeville, PA, USA); gas chromatography mass spectroscopy (GCMS), model MS QP5050A; Shimadzu, Tokyo, Japan; and NMR spectra (^1^H and ^13^C) were obtained by using a JEOL FTNMR 500 MHz transformed spectrometer in CDCl_3_ or CD_3_OD and using tetramethylsilane (TMS) as the internal standard 500 MHz for both ^1^H NMR and ^13^C NMR, respectively.

(3) $$Degree\;of\;Conversion\%=\frac{V_{control}-V_{test}}{V_{control}}\times 100$$

In which $V_{test}$ and $V_{control}$ are NaOH (0.1 M) volumes used for neutralizing the excess acid in the test (with enzyme) and control (without enzyme) experiments, respectively.

#### 3.2.2. Antibacterial Assay

The antibacterial effect of azelaic acid derivatives (dilaurylazelate ester) against *Staphylococcus epidermidis S273* were studied by means of agar diffusion method. The test is carried out by placing 6 mm diameter of paper disc containing antibiotic onto a plate on which microbes are growing. The microbe culture is standardized to 0.5 McFarland standard, which is approximately 10^8^ cells (Mueller Hinton agar pH 7.3). Phosphate buffer (NaH_2_PO_4_) was added until pH 5.6. Streptomycin standard was used for each bacteria. The plates are inverted and incubated at 30–37°C for 18–24 h using a target organism. After incubation, zone of inhibition appeared and each plate was examined. The diameters of zones of complete inhibition are measured including the diameter of disc. Zones are measured to the nearest whole millimeter using sliding calipers, which are held on the back of the inverted petri plate.

#### 3.2.3. Cytotoxicity Assay

Azelaic acid and azelaic acid derivative (dilaurylazelate ester) were tested further for their cytotoxicity activity. Both of the samples were tested on 3T3 normal fibroblast cells. All cells were maintained in RPMI medium supplemented with 10% fetus calf serum (FCS), 100 unit/mL penicillin, and 0.1 mg/mL streptomycin. Initially, cell culture with a concentration of 1 × 105 cells/mL was prepared and plated (100 μL/well) onto 96-wellplates. The cell was incubated for 24 h at 37°, 5% CO_2_. After incubation, the diluted ranges of samples (1.56, 3.125, 6.25, 12.5, 25, 50, 100 and 120 μg/mL) were added to each well and incubated for another 24 h. Then, MTT solution at 2 mg/mL was added to each well and left to stand for 1 h before being measured at 570 nm. The cytotoxicity result was expressed as IC_50_, defined as the concentration of sample that caused inhibition of 50% cell growth.

## 4. Conclusions

In general, the optimization of a lipase (Novozym 435) catalyzed esterification reaction to produce dilaurylazelate ester by applying response surface methodology was successfully carried out. The effect of four main reaction parameters (reaction times, amounts of enzyme, reaction temperatures, and substrate molar ratio) were evaluated using an empirical model. The coefficient of the determination value (R^2^) of 0.9732 was desirable and demonstrated a good fit with the experimental data. This fitted model could be applied to find the yields of production of dilaurylazelate ester under any given conditions within the experimental range. Experimentally, a high percentage of conversion (95.38%) was achieved, which also matched well with the predicted value of 96.23%, by applying only a minimal enzyme amount. Therefore, the scaled-up enzymatic production of dilaurylazelate ester, taking into consideration the economical and environmental aspects, can be carried out in the future by applying the RSM techniques. In addition, the cytotoxicity was tested on 3T3 normal fibroblast cells; the results showed that dilaurylazelate ester is non-toxic compared with AzA and safe to be used in pharmaceutical applications, and it has interesting antibacterial properties which are worthy of further investigation in clinical trials of a relevant group of test subjects.

## Figures and Tables

**Table 1:** Variables and their levels employed in the central composite rotatable design.

Variables		Units	Coded Level of Variables				
			−2	−1	0	1	2
X1	Enzyme amount	gram	0.05	0.15	0.25	0.35	0.45
X2	Reaction time	min	90	180	270	360	450
X3	Reaction temperature	°C	40	46	52	58	64
X4	Molar ratio of substrates	AA:LA (mole:mole)	1:3	1:4.5	1:6	1:7.5	1:9

**Table 2:** Central composite rotatable design matrix and result for the model of dilaurylazelate ester synthesis.

Run No.	Enzyme Amount (g)	Reaction Time (min)	Reaction Temperature (°C)	Molar Ratio of Substrates, AzA:LA (mole)	Degree of Conversion %	
					Actual Value	Predicted Value
1	0.15	180	58	1:4.5	87.83	87.82
2	0.15	360	58	1:4.5	93.51	92.40
3	0.25	270	52	1:6	86.61	86.24
4	0.35	180	58	1:7.5	87.89	87.60
5	0.25	90	52	1:6	80.80	81.19
6	0.35	180	46	1:4.5	80.62	80.59
7	0.35	360	58	1:4.5	95.22	96.21
8	0.25	270	52	1:9	79.69	79.28
9	0.25	270	52	1:6	87.95	86.24
10	0.35	360	46	1:4.5	95.40	94.88
11	0.35	360	46	1:7.5	90.83	89.84
12	0.25	450	52	1:6	96.74	96.49
13	0.15	360	58	1:7.5	86.73	87.36
14	0.25	270	40	1:6	85.71	86.60
15	0.35	360	58	1:7.5	90.69	91.16
16	0.25	270	52	1:6	85.27	86.24
17	0.15	180	46	1:7.5	83.91	82.50
18	0.25	270	52	1:3	85.24	85.79
19	0.35	180	46	1:7.5	78.76	79.12
20	0.25	270	52	1:6	84.38	86.24
21	0.15	360	46	1:4.5	95.38	95.71
22	0.05	270	52	1:6	93.20	93.04
23	0.15	180	58	1:7.5	84.98	86.35
24	0.25	270	52	1:6	86.61	86.24
25	0.15	360	46	1:7.5	90.13	90.67
26	0.25	270	64	1:6	92.52	91.77
27	0.35	180	58	1:4.5	89.78	89.07
28	0.15	180	46	1:4.5	84.14	83.96
29	0.25	270	52	1:6	86.61	86.24
30	0.45	270	52	1:6	93.17	93.47

**Table 3:** Analysis of variance (ANOVA) for the quadratic model developed for synthesis of dilaurylazelate ester.

Source	Sum of Squares	DF *	Mean Square	*F*-Value	*p*-Value Prob > F
Model	688.58	12	57.38	51.37	<0.0001
A-Novozym 435 amount	0.27	1	0.27	0.24	0.6308
B-Reaction time	351.47	1	351.47	314.67	<0.0001
C-Reaction temperature	40.23	1	40.23	36.02	<0.0001
D-Molar ratio of substrates	63.52	1	63.52	56.87	<0.0001
AB	6.52	1	6.52	5.84	0.0272
AC	21.40	1	21.40	19.16	0.0004
BC	51.28	1	51.28	45.92	<0.0001
BD	12.77	1	12.77	11.43	0.0035
A2	84.45	1	84.45	75.60	<0.0001
B2	11.62	1	11.62	10.40	0.0050
C2	14.91	1	14.91	13.35	0.0020
D2	23.47	1	23.47	21.01	0.0003
Residual	18.99	17	1.12	-	-
Lack of fit	11.25	12	0.94	0.61	0.7794
Pure error	7.74	5	1.55	-	-
Standard deviation		1.06			
PRESS		54.47			
R2		0.9732			
Adjusted R2		0.9542			
Predicted R2		0.9230			
Coefficient of variation		1.20			
Adequate Precision		24.973			

* DF: Degree of Freedom.

**Table 4:** Optimum conditions derived by response surface methodology (RSM) and validation set for synthesis of dilaurylazelate ester.

	Enzyme Amount (gram)	Independent Variables			Conversion%		
		Reaction Time (min)	Reaction Temperature (°C)	Molar Ratio of Substrates, AA:LA (mole)	Actual Value	Predicted Value	RSE (%)
Validation Set	0.20	180	50	1:6	84.23	83.01	1.47
	0.30	300	52	1:5	91.19	89.05	2.40
	0.20	360	52	1:6.5	90.99	89.84	1.28
	0.16	250	55	1:5.5	87.17	87.75	0.66
	0.10	360	52	1:6	95.10	93.54	1.66
Optimal conditions	0.14	360	46	1:4.1	95.38	96.23	0.88

**Table 5:** The size of the inhibition zone indicated the antibacterial effect of AzA and dilaurylazelate ester on bacteria *Staphylococcus epidermidis S273.*

Samples	Diameter Zone Inhibition (mm)on Bacteria *Staphylococcus epidermidis S273*
Azelaic acid	11.5 ± 0.1 mm
Dilauryl azelate ester	9.0 ± 0.1 mm
Standard (Streptomycin)	28.0 ± 0.1 mm

## References

[1] Kosmadaki M., Katsambas A. (2017). Topical treatments for acne. Clin. Dermatol..

[2] Khairudin N., Basri M., Masoumi H.R.F., Samiun W.S., Samson S. (2015). Lipase-catalyzed synthesis of dilauryl azelate ester: Process optimization by artificial neural networks and reusability study. RSC Adv..

[3] Al-Marabeh S., Khalil E., Khanfar M., Al-Bakri A.G., Alzweiri M. (2016). A prodrug approach to enhance azelaic acid percutaneous availability. Pharm. Dev. Technol..

[4] Tamarkin D. (2004). Novel Conjugate Compounds and Dermatological Compositions Thereof. U.S. Patent.

[5] Manosroi A., Panyosak A., Rojanasakul Y., Manosroi J. (2007). Characteristics and anti-proliferative activity of azelaic acid and its derivatives entrapped in bilayer vesicles in cancer cell lines. J. Drug Target..

[6] Fang J.-Y., Leu Y.-L. (2006). Prodrug strategy for enhancing drug delivery via skin. Curr. Drug Discov. Technol..

[7] Svensson C.K. (2009). Biotransformation of drugs in human skin. Drug Metab. Dispos..

[8] Prausnitz M.R., Langer R. (2008). Transdermal drug delivery. Nat. Biotechnol..

[9] Hsieh P.-W., Al-Suwayeh S.A., Fang C.-L., Lin C.-F., Chen C.-C., Fang J.-Y. (2012). The co-drug of conjugated hydroquinone and azelaic acid to enhance topical skin targeting and decrease penetration through the skin. Eur. J. Pharm. Biopharm..

[10] Narula O. (1995). Treatise on Fats, Fatty Acids & Oleochemicals.

[11] Cao L., Fischer A., Bornscheuer U.T., Schmid R.D. (1996). Lipase-catalyzed solid phase synthesis of sugar fatty acid esters. Biocatal. Biotransform..

[12] Chaibakhsh N., Abdul Rahman M.B., Basri M., Salleh A.B., Rahman R.N.Z.R.A. (2009). Effect of alcohol chain length on the optimum conditions for lipase-catalyzed synthesis of adipate esters. Biocatal. Biotransform..

[13] Syamsul K., Salina M., Siti S., Hanina M., Basyaruddin M., Jusoff K. (2010). Green synthesis of lauryl palmitate via lipase-catalyzed reaction. World Appl. Sci. J..

[14] Zisis T., Freddolino P.L., Turunen P., van Teeseling M.C., Rowan A.E., Blank K.G. (2015). Interfacial activation of *Candida antarctica* lipase b: Combined evidence from experiment and simulation. Biochemistry.

[15] Anderson E.M., Larsson K.M., Kirk O. (1998). One biocatalyst—Many applications: The use of *Candida antarctica* b-lipase in organic synthesis. Biocatal. Biotransform..

[16] Ashari S.E., Mohamad R., Ariff A., Basri M., Salleh A.B. (2009). Optimization of enzymatic synthesis of palm-based kojic acid ester using response surface methodology. J. Oleo Sci..

[17] Khandanlou R., Ahmad M.B., Masoumi H.R.F., Shameli K., Basri M., Kalantari K. (2015). Rapid adsorption of copper (ii) and lead (ii) by rice straw/fe 3 o 4 nanocomposite: Optimization, equilibrium isotherms, and adsorption kinetics study. PLoS ONE.

[18] Sohrabi M.R., Amiri S., Masoumi H.R.F., Moghri M. (2014). Optimization of direct yellow 12 dye removal by nanoscale zero-valent iron using response surface methodology. J. Ind. Eng. Chem..

[19] Yetilmezsoy K., Demirel S., Vanderbei R.J. (2009). Response surface modeling of pb (ii) removal from aqueous solution by *Pistacia vera* L.: Box—Behnken experimental design. J. Hazard. Mater..

[20] Krishna S.H., Divakar S., Prapulla S.G., Karanth N.G. (2001). Enzymatic synthesis of isoamyl acetate using immobilized lipase from rhizomucor miehei. J. Biotechnol..

[21] Radzi S.M., Basri M., Salleh A.B., Ariff A., Mohamad R., Abdul Rahman M.B., Abdul Rahman R.N.Z. (2005). Large scale production of liquid wax ester by immobilized lipase. J. Oleo Sci..

[22] Basyaruddin A.R.M., Chaibakhsh N., Basri M., Rahman R.N.Z.R.A., Salleh A.B., Radzi S.M. (2008). Modeling and optimization of lipase-catalyzed synthesis of dilauryl adipate ester by response surface methodology. J. Chem. Technol. Biotechnol..

[23] Hari Krishna S., Sattur A.P., Karanth N.G. (2001). Lipase-catalysed synthesis of isoamly isobutyrate-optimisation using a central composite rotatable design. Process Biochem..

[24] Masoumi H.R.F., Kassim A., Basri M., Abdullah D.K., Haron M.J. (2011). Multivariate optimization in the biosynthesis of a triethanolamine (tea)-based esterquat cationic surfactant using an artificial neural network. Molecules.

[25] Ngan C.L., Basri M., Lye F.F., Fard Masoumi H.R., Tripathy M., Abedi Karjiban R., Abdul-Malek E. (2014). Comparison of box—Behnken and central composite designs in optimization of fullerene loaded palm-based nano-emulsions for cosmeceutical application. Ind. Crop Prod..

[26] Gad S.C., Ballantyne B., Marvis Y., Turner P. (2009). Alternatives to in vivo studies in toxicology. General, Applied and Systems Toxicology.

[27] Chaibakhsh N., Rahman M.B.A., Abd-Aziz S., Basri M., Salleh A.B., Rahman R.N.Z.R.A. (2009). Optimized lipase-catalyzed synthesis of adipate ester in a solvent-free system. J. Ind. Microbiol. Biotechnol..

[28] Fard Masoumi H.R., Basri M., Kassim A., Kuang Abdullah D., Abdollahi Y., Abd Gani S.S., Rezaee M. (2013). Statistical optimization of process parameters for lipase-catalyzed synthesis of triethanolamine-based esterquats using response surface methodology in 2-liter bioreactor. Sci. World J..

